# On the Effect of High Stimulation Rates on Temporal Loudness Integration in Cochlear Implant Users

**DOI:** 10.1177/23312165231207229

**Published:** 2023-11-08

**Authors:** Miguel Obando-Leitón, Anna Dietze, Carmen M. Castañeda González, Ali Saeedi, Sonja Karg, Werner Hemmert

**Affiliations:** 1 Bio-Inspired Information Processing, Munich Institute of Biomedical Engineering, 9184 Technical University of Munich, Garching, Germany; 2 TUM School of Computation, Information and Technology, 9184 Technical University of Munich, Munich, Germany; 3 Graduate School of Systemic Neurosciences, 9183 Ludwig-Maximilians Universität München, Planegg, Germany; 4 Munich Institute of Robotics and Machine Intelligence, 9184 Technical University of Munich, Munich, Germany

**Keywords:** cochlear implants, temporal loudness integration, pulse rate dependencies, multi-pulse integration, psychophysics in humans

## Abstract

Long stimuli have lower detection thresholds or are perceived louder than short stimuli with the same intensity, an effect known as temporal loudness integration (TLI). In electric hearing, TLI for pulse trains with a fixed rate but varying number of pulses, i.e. stimulus duration, has mainly been investigated at clinically used stimulation rates. To study the effect of an overall effective stimulation rate at 100% channel crosstalk, we investigated TLI with (a) a clinically used single-channel stimulation rate of 1,500 pps and (b) a high stimulation rate of 18,000 pps, both for an apical and a basal electrode. Thresholds (THR), a line of equal loudness (BAL), and maximum acceptable levels (MALs) were measured in 10 MED-EL cochlear implant users. Stimulus durations varied from a single pulse to 300 ms long pulse trains. At 18,000 pps, the dynamic range (DR) increased by 7.36±3.16 dB for the 300 ms pulse train. Amplitudes at THR, BAL, and MAL decreased monotonically with increasing stimulus duration. The decline was fitted with high accuracy with a power law function ( $R^{2}=0.94\pm 0.06$ ). Threshold slopes were −1.05±0.36 and −1.66±0.30 dB per doubling of duration for the low and high rate, respectively, and were shallower than for acoustic hearing. The electrode location did not affect the amplitudes or slopes of the TLI curves. THR, BAL, and MAL were always lower for the higher rate and the DR was larger at the higher rate at all measured durations.

## Introduction

Signal processing in cochlear implants (CIs) assumes a close relation between stimulation amplitude and perceived loudness. Understanding the exact mechanisms and the influences of different parameters will help find a superior approach to loudness coding. This study investigated how temporal loudness integration (TLI) in CIs is affected by stimulation rate, stimulation level, and electrode location.

In acoustic hearing, TLI describes either the decrease in detection thresholds or the increase in perceived loudness of suprathreshold stimuli, when the stimulus duration is increased up to a critical duration of about 150 ms (Stevens & Hall, 1966; Ogura et al., 1991). By now, TLI measurements have been done with many species, including humans, primates, carnivores, birds, and even fish (reviewed by Heil et al., 2017). The summary revealed—besides interindividual differences—striking similarities in the overall shape of these TLI curves. A power-law function describes the relationship between threshold and time very well for acoustic hearing. This function has a slope of about −2 dB per doubling of duration or approximately −20∕3 dB per tenfold increase of duration (decade).

TLI in electric hearing refers to the observed reduction of detection thresholds, or alternatively, the necessary amplitude reduction of suprathreshold stimuli when increasing the stimulus duration while maintaining a fixed stimulation rate and phase duration. Compared with acoustic stimulation, direct electrical stimulation with a CI allows stimulation paradigms that go beyond what is possible for acoustic signals. First, in electric hearing, stimulation durations as short as a single electric pulse can be applied without spectral broadening. Second, the stimulation rate can be changed without affecting the location of the excitation pattern. While it is assumed that the same central processing takes place in acoustic and electric hearing, only with different neural inputs, slopes of TLI curves are much shallower with electric stimulation, e.g. −0.42 dB per doubling of stimulus pulses (Donaldson et al., 1997) and −1.5 dB per tenfold increase in duration, equivalent to a slope of −0.45 dB per doubling of duration (Gerken et al., 1991). However, direct comparisons between normal-hearing and CI TLI slopes should be made with caution considering the difference in dynamic ranges (DRs) (about 100 dB in acoustic hearing and around 10 dB in electric hearing). Moreover, TLI curves in CI users show a high variability across subjects, and in some cases, across electrodes in a single subject’s cochlea (Donaldson et al., 1997; Shannon, 1990). There have been different attempts to describe and explain the amplitude vs. duration relationship in electric hearing, including models by Shannon (1989), Carlyon et al. (2005), and McKay and McDermott (1998).

A reduction in electric detection thresholds, or increase in the perceived loudness of suprathreshold stimuli, has been observed not only when increasing the pulse train duration while keeping a fixed stimulation rate and phase duration (TLI) but also when increasing the stimulation rate while maintaining the pulse train duration and phase duration fixed. This effect of different pulse rates on the hearing threshold was also termed multi-pulse integration (MPI) in previous investigations (Carlyon et al., 2015; Kreft et al., 2004; Pfingst et al., 2011; Zhou et al., 2015). Carlyon et al. (2015) found a decrease in threshold of 2.7 dB per doubling of the rate between 500 and 3,500 pps for 400 ms pulse trains. A decrease of 2.4 dB and 1.2 dB per doubling of the rate for threshold and maximum acceptable levels (MALs), respectively, was found by Kreft et al. (2004) (200 ms pulse trains) between 200 and 6,500 pps.

A neuron between two electrodes is stimulated by the pulses from both electrodes, which follow at a rate of rate × electrodes. If we assume a broad current spread, then also farther electrodes will contribute to the nuerons’ stimulation, which results in a high-frequency burst of stimulation. Studies in a human cadaver cochlea (Ifukube & White, 1987) and electrical cochlear models (e.g Bai et al., 2019; Malherbe et al., 2016) have shown a broad current spread of stimulation throughout the whole cochlea. Very broad excitation regions have also been found in form of flat spatial tuning curves or tuning curves with “extended tails” in some subjects (Nelson et al., 2008, 2011). Hence, the actual stimulation rate of a single neuron might be up to the stimulation rate of a single electrode multiplied by the number of electrodes in use. The single-channel stimulation rate is usually set to approximately 1,500 pps in MED-EL implants, in which 12 electrodes are available. We therefore selected 18,000 pps as the highest rate spiral ganglion neurons are subjected to.

In this study, we focus on the shape of threshold and suprathreshold curves of equal loudness as a function of stimulus duration. All our measurements relate to the psychophysical measurement of loudness. Therefore, our results describe the effects of TLI in the auditory pathway. We were interested in the characteristics of TLI in CIs, as different peripheral mechanisms are in place compared to normal (acoustic) hearing. In particular, we investigated how TLI curves between a single pulse and 300 ms are affected by stimulation rate, stimulation level, and electrode location. TLI curves were studied at a low (1,500 pps) and a high (18,000 pps) stimulation rate. To the best of our knowledge, TLI curves have not been obtained for stimulation rates as high as the one used here. Additionally, we asked to which extent a simple power-law function is suitable to model electric hearing data up to moderate durations and at different stimulation levels. Power-law functions are frequently used to accurately describe TLI curves in acoustic hearing as well as in other sensory modalities such as electrocutaneous sensation (Higashiyama & Tashiro, 1983; Tashiro & Higashiyama, 1981). Moreover, it has been observed that the amount of TLI varies with stimulation level in normal hearing listeners (Buus et al., 1997; Florentine et al., 1996; Garnier et al., 1999) and listeners with cochlear hearing loss (Buus et al., 1999). To this end, in addition to TLI curves at threshold (THR), we also measured TLI curves at MALs and at a line of equal loudness (BAL) between THR and MAL at an apical and a basal electrode, respectively.

## Methods

### Subjects

Ten subjects ( M=54 years, SD=15 years; 3 male, 7 female) with CIs from MED-EL participated in our study. Subjects S1 and S8 agreed to do the experiments with both ears for a total of 12 measured ears. Details are presented in Table 1.

**Table 1.** Demographic Information of Subjects.

Subject	Age (years)	Gender	HL onset	Etiology	CI use (months)	CI Model
S1r	65	F	40	Unknown	60	Sonata
S1l	65	F	40	Unknown	54	Sonata
S2	23	M	1	Meningitis	120	Sonata
S3	53	F	30	Hereditary	28	Synchrony
S4	78	M	58	Acute HL	148	Pulsar
S5	42	F	1	Cholesteatoma	36	Synchrony
S6	55	M	5	Otitis	72	Concerto
S7	42	F	Birth	Acute HL	60	Concerto
S8r	64	F	35	Meningitis	90	Concerto
S8l	64	F	27	Meningitis	30	Synchrony
S9	60	F	9	Otitis	72	Concerto
S10	59	F	30	Unknown	132	Pulsar

*Note*. HL = hearing loss; CI = cochlear implant.

All subjects gave their informed written consent for their participation and received monetary compensation. Measurements were conducted in accordance with the Declaration of Helsinki and approved by the medical ethics committee of the Klinikum rechts der Isar (Munich, 2126/08).

### Equipment

The core of the experimental setup was a computer equipped with a digital card (Model NI PCIe-6361, National Instruments, Austin, Texas, USA). Pulse trains were created by sending all parameters (phase and gap duration, stimulation rate, pulse train duration, and stimulation amplitude) to the Research Interface Box 2 (RIB2, Institute of Ion Physics and Applied Physics, University of Innsbruck). The RIB2 turns the given information into pulses that are then sent out directly to the implanted parts of a CI. This setup allowed for full control over the stimulation, bypassing the sound processor. Stimuli were checked with a detector box (Institute of Ion Physics and Applied Physics, University of Innsbruck) that emulates a Pulsar implant and was connected to a digital oscilloscope. Subjects responded to the stimuli either by using a computer mouse to click response buttons of a graphical user interface (GUI) on the computer screen or by pressing the respective buttons on a computer keyboard.

Python (Version 2.7, 32-bit) was used to create the stimulation pulses and adapt them in real time, corresponding to the subjects’ responses. For data analysis, MATLAB (Version 9.10.0.1739362, R2021a Update 5, The MathWorks Inc., Natick, Massachusetts) with the Curve Fitting Toolbox (Version 3.5.13, The MathWorks, Inc.) was used. Statistical analyses were performed on IBM SPSS Statistics (Version 28.0.0.0 (190)) and R (The R Foundation for Statistical Computing, R version 4.2.2, “Superpower” Package), also available as an online tool (Superpower’s Power Shiny App, https://shiny.ieis.tue.nl/anova˙power/) developed by  Lakens and Caldwell (2021).

### Stimuli

For all measurements, monopolar stimulation was applied. Biphasic, charge-balanced pulses with leading cathodic (negative) phase were used. To allow for very high stimulation rates, the phase duration was set to 23.33 µs with a minimally available gap of 2.10 µs between the phases. Stimuli differed in stimulation electrode, rate, and duration (or equivalently, the number of pulses). A stimulation amplitude of 1,200 CU (62 dB re 1 CU) is set as an upper limit for stimulation by both the RIB2 software and the CI. 1 CU is roughly equivalent to 1 µA. Compliance limits were calculated individually in case the selected electrode’s impedances exceeded 5.6 kΩ. The compliance limit for MED-EL devices is 6.8 V. For example, for an impedance of 10 kΩ, the compliance limit is 680 µA. All stimuli were within the safe limits of stimulation. The maximally delivered charge was 27 nC.

In all experiments, the presented pulse trains were separated by a fixed silent gap of 500 ms. Since the longest duration used was 300 ms, the effect of forward masking was presumably reduced to a minimum after the pause. Nelson and Donaldson (2002) found average time constants of 54 ms for the exponential decay of masking after a 320 ms long masker with a frequency of 500 Hz. Even with the largest time constant they found (163 ms), less than 5 % threshold shift would be observed for a 10 or 30 ms probe pulse train after a pause of 500 ms. Regarding the effect of stimulation rate on forward masking time constants, no studies investigated rates as high as the ones we used in this study. However, Adel et al. (2017) showed that masking by pulse trains of high stimulation rate (5,000 pps) was even less pronounced than masking induced by low-rate (250 pps) pulse train maskers, when presented at the same loudness, as measured through the shift in probe detection thresholds 16 ms after the masker offset.

#### Electrodes

The same measurements were done at two different electrodes on the array. If not hindered by any reason, electrodes 3 (apical) and 10 (basal) were selected out of 12 electrodes in MED-EL CIs. Otherwise, neighboring electrodes were chosen. The selected electrodes had impedances lower than 10 kΩ. Compliance limits were calculated individually for electrodes with impedances larger than 5.6 kΩ.

#### Stimulation Rates

Two stimulation rates were used. The lower stimulation rate (1,500 pps) represented a typical stimulation rate present at a single contact of the electrode array in normal CI settings (without channel crosstalk). The higher rate (18,000 pps) was chosen to investigate the effect of an increased overall effective stimulation rate of 12 times the single-channel stimulation rate (at 100 % channel crosstalk).

#### Duration

At the higher rate, stimuli with the number of pulses ranging from 1 to 5400 were presented. For the lower rate, the stimuli consisted of 1 pulse to 450 pulses. The longest investigated pulse trains had thus a duration of 300 ms. The maximum duration was chosen as this is approximately the duration of a syllable, and also because it is commonly used as pulse train duration in clinical fittings (Wolfe & Schafer, 2014). Table 2 offers an overview of all tested rate × duration conditions and their corresponding number of pulses. The single pulse condition was only measured once and later assigned to both stimulation rates for the analyses. It is not possible to assign a train-duration to a stimulation with single pulses unambiguously, however, pulse trains can be labeled by their number of pulses. Therefore, no durations in milliseconds are shown in Table 2 for single pulses. The number of pulses was selected as the x -axis (upper axis), whereas the time axis (lower axis) had to be cut to accommodate the single pulse condition.

**Figure 1.**
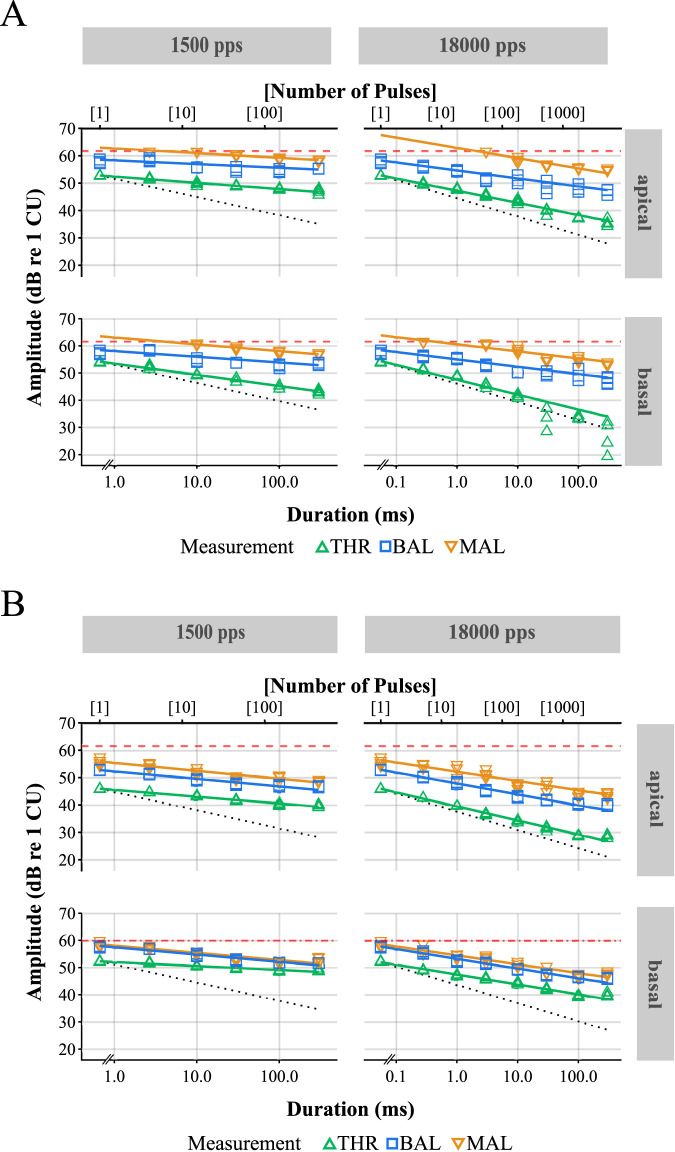
Exemplary data obtained from subject S1l (A) and S7 (B) for THR, MALs, and loudness balancing (BAL). The solid lines depict power functions (equation 2) fitted to the data points. The dotted lines show the TLI slopes of −20∕3 dB per decade in normal hearing. In case the electrode’s impedance was below 5.6 kΩ, the dashed lines represent the maximum possible stimulation amplitude of 1,200 CU. Else, the individual compliance limit is denoted by the dashdotted line. Top: Apical electrode. Bottom: Basal electrode. Left: 1,500 pps. Right: 18,000 pps. MAL = maximum acceptable level; THR = threshold; TLI = temporal loudness integration.

**Figure 2.**
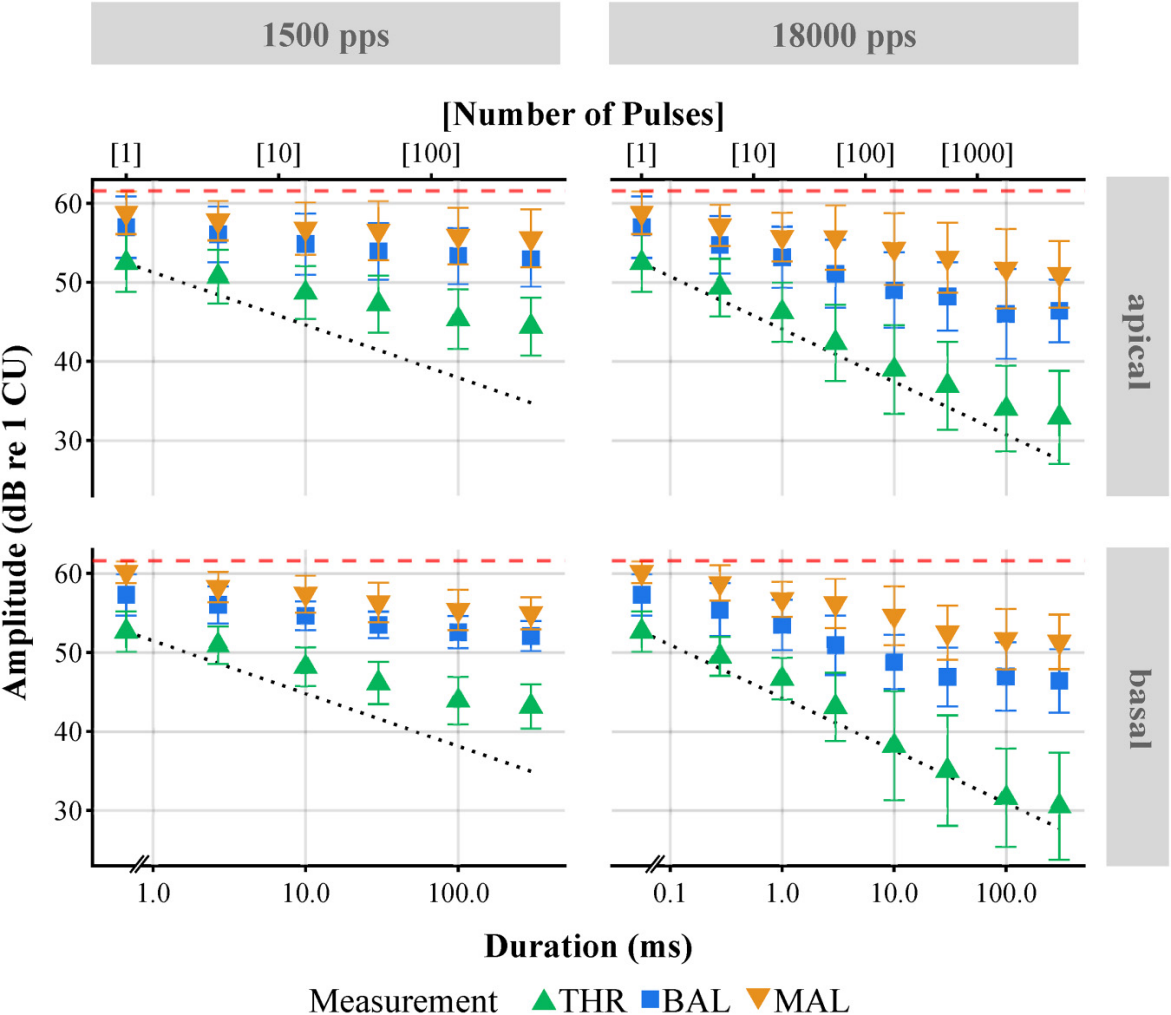
Mean amplitudes as a function of duration for THR, MALs, and loudness balancing (BAL) for all subjects. The dotted lines show the TLI slopes of −20∕3 dB per decade in normal hearing. The dashed lines represent the maximum possible stimulation amplitude of 1,200 CU. Symbols represent the mean over all subjects. Error bars indicate one standard deviation from the mean. Top: Apical electrode. Bottom: Basal electrode. Left: 1,500 pps. Right: 18,000 pps. MAL = maximum acceptable level; THR = threshold; TLI = temporal loudness integration.

**Table 2.** Stimulation Durations and Their Corresponding Number of Pulses (In Brackets) Used at the Two Stimulation Rates.

Rate (pps)	Duration (ms) [Number of pulses]							
1,500				2.67	10	30	100	300
			[1]	[4]	[15]	[45]	[150]	[450]
18,000		0.28	1	3	10	30	100	300
	[1]	[5]	[18]	[54]	[180]	[450]	[1800]	[5400]

### Experimental Procedure

The experiment consisted of three parts. In the first part, current amplitudes corresponding to threshold (THR) were determined. For the second part, subjects adjusted the stimulus levels to the maximum acceptable amplitude, referred to as MAL. The third part consisted of a loudness balancing procedure (BAL). The method of adjustment, in which subjects increased and decreased the stimulation amplitude to the desired value by themselves, was used in all THR, BAL, and MAL measurements, including training trials.

A GUI created with Python was displayed to the subjects. Buttons to increase or reduce the stimulation amplitude in large and small steps were visible, as well as a button to save the adjusted amplitudes. All responses could also be entered via a computer keyboard. The corresponding keys were color-matched to those on the screen. Changing the amplitude in small steps caused an increase or decrease of the current level by 1.18 to 9.45 CU, depending on the amplitude. The large steps changed the level by ±18.90 CU, with one exception. In training trials starting at zero amplitude, large steps increased the current amplitude by 28.35 CU, up until the first reversal. During the training phases of THR and MAL, a representation of the chosen amplitudes was visible to provide feedback for the subjects. No feedback regarding the chosen current amplitude was given outside of training. Throughout the experiment, pauses were automatically initiated every 20 minutes if subjects did not ask for a pause before.

#### Thresholds

A training phase made sure that the subjects understood the task and familiarized themselves with the setup. From the training trials, which were not used for the experiment, preliminary THR estimates were acquired. Preliminary THRs were measured at three well-separated durations (four at the higher rate) in the duration-threshold curve for each electrode × rate combination. From these points, a first estimation of the THR was obtained by linear interpolation in the log-log representation of the duration-threshold pairs. In the training phase, the starting current amplitude was always zero. The resulting estimates were used in the following measurements to speed-up the time needed to reach THR.

THRs were measured four times for all possible electrode × rate × duration combinations. The starting points for the adjustment by the subjects varied randomly between 80 % and 90 % or 110 % and 120 % of the preliminary THR estimates determined during the training phase. Care was taken that two of the starting points were above and two below the THR estimate. These variations were unknown to the subject and aimed to reduce biases induced by the starting point and the direction from which the threshold is reached (Gelfand, 2017). From the starting points, subjects adjusted the perceived loudness by increasing and decreasing the current amplitudes until the stimulus was just barely audible. They were encouraged to use the larger step buttons first to reach the vicinity of the threshold fast and then use the smaller step changes for fine adjustments. To further reduce biases, they were asked to bracket their thresholds (i.e. reach them from above and below) before saving the response (Gelfand, 2017). In cases where the result was out of range (above compliance limit) twice, the remaining two blocks of this condition were skipped and left blank for analyses. All electrode × rate × duration combinations were presented in a randomized order. Randomization was not applied to the training measurements.

#### Maximum Acceptable Levels

Analogously to the THR measurements, a short training phase ensured that every subject understood the task. During training, preliminary MALs were measured only for the longest pulse train duration (300 ms) for a first estimation of the DR.

MALs were measured four times for all possible electrode × rate × duration combinations. Starting from just below 50 % of the estimated DR, subjects adjusted the perceived loudness by increasing (and decreasing if needed) the current amplitude until the stimulus was very loud but still acceptable over a longer time. They were encouraged to use the larger steps first to reach the desired amplitude fast and then use the smaller step changes for fine adjustments to the MAL. If MAL was not reached (before arriving at the compliance limit) twice, further repetitions of this duration were skipped and left blank for analyses. MALs were measured randomized over all electrode × rate × duration conditions. Randomization was not applied to the training measurements.

#### Curve of Equal Loudness

In the third part of the experiment, a loudness balancing (BAL) procedure as a function of stimulus duration for each of the 4 electrode × rate conditions, yielded a total of four BAL curves. For this experimental part, the graphical interface was extended by two visual displays that flashed in gray and yellow at the same time the fixed stimulus and the probe stimulus were presented. This was done to indicate which of the two stimuli was fixed (gray) and for which the amplitude could be adjusted by the subject (yellow). Subjects were instructed to adjust the amplitude of the probe stimulus until its loudness matched the loudness of the fixed stimulus. Colors were chosen such that they were perceived on the computer screen with approximately the same luminance.

**Figure 3.**
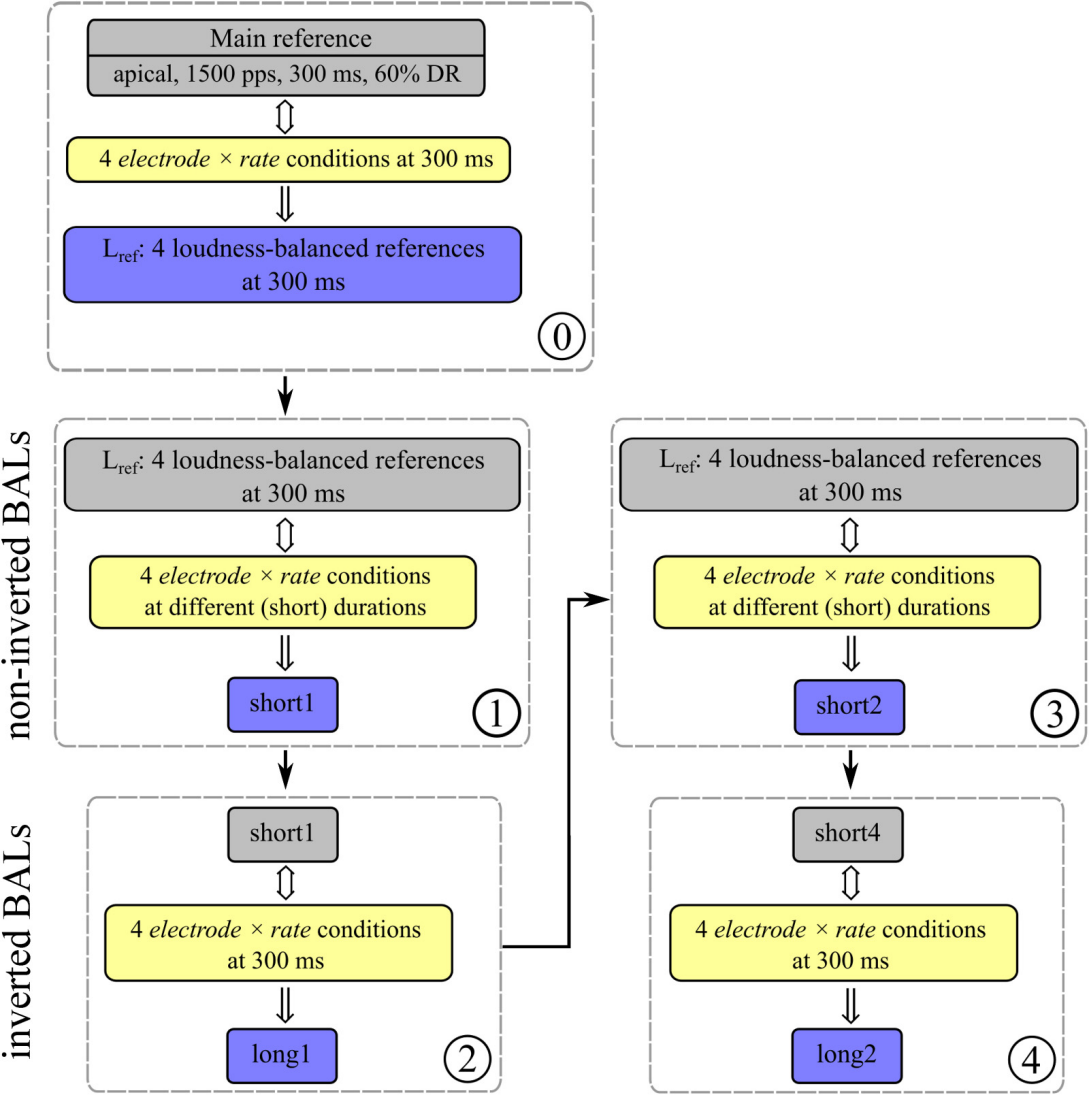
Flowchart depicting the entire loudness balancing procedure. Within each step (dashed box), the first box represents the fixed stimulus, the second box the probe, and the third box the result of the balancing procedure. The order of the balancing procedure is numbered at the lower-right corner of each dashed box (from 0 to 4). At step 0, all 300 ms-references were balanced against the loudness of a main reference stimulus presented with 1,500 pps, 300 ms at 60% of its dynamic range (DR) at the apical electrode. Steps 1 and 3 show the noninverted BALs, where the loudness of the shorter stimuli had to be balanced to the corresponding previously-adjusted 300 ms reference stimuli $L_{ref}$ yielding the amplitudes short1 and short2 . Steps 2 and 4 show the inverted BALs. The 300 ms (long) stimuli served as probes and were compared to the fixed amplitudes of the shorter stimuli ( short1 and short4 ) resulting in the amplitudes long1 and long2 . The calculation for the amplitude of the fixed stimulus *short4* was based on equation 1 using the results from the noninverted BALs (steps 1 and 3).

A flowchart depicting the entire loudness balancing procedure is shown in Figure 3. Foremost (⓪), all 4 electrode × rate conditions at 300 ms were balanced to the loudness of a main reference stimulus presented with 1,500 pps, 300 ms at the apical electrode, fixed to 60 % of its DR. This led to a set of four 300 ms long loudness-balanced reference stimuli ( $L_{\text{\_\{ref\}}}$ ), one for each electrode × rate condition. Right afterwards, subjects were presented with all four 300 ms reference stimuli $L_{\text{ \_\{ref\}}}$ in a row. As they had been balanced before, all of them should appear at the same loudness. In case this was not true, subjects were able to change the amplitude of individual signals (making them either louder or softer in small step sizes) to equalize them in terms of loudness. The purpose of this balancing step was to represent an equal loudness across all BAL measurements, which allowed for valid comparisons across all four BAL curves later on.

After having balanced the four 300 ms-references $L_{ref}$ , the experiment continued with balancing the loudness of two signals of different duration (stimulus with duration smaller than 300 ms (short) vs. stimulus with duration equal to 300 ms (long)). For each of the 4 electrode × rate conditions, all possible short vs. long comparison pairs were balanced four times each (Figure 3, blocks ① – ④). In two of the four balancings (first ① and third ③), the stimulus that was fixed in amplitude was the corresponding previously-balanced 300 ms-reference $L_{\text{\_\{ref\}}}$ . The probe (the adjustable stimulus) was the stimulus with a duration smaller than 300 ms (short). These were the so-called noninverted balancing trials, which led to the resulting amplitudes *short1* (①) and *short2* (③). In the other two balancings (second ② and fourth ④), the 300 ms stimulus (long) had to be adjusted to match the loudness of the stimulus with duration smaller than 300 ms (short). These were the so-called inverted balancings, resulting in the amplitudes *long1* (②) and *long2* (④). The amplitude of the short (fixed) stimulus for the second ② balancing was set to *short1* (①). The amplitude of the fixed stimulus *short4* for the fourth ④ balancing measurement was calculated based on equation 1 (Adel et al., 2017; McKay et al., 2003), using the results ( short1 and short2 ) from the noninverted BALs (① and ③). Equation 1 describes the average of the differences d (in CU) between amplitudes of short and long stimuli in the inverted and noninverted blocks, added to the amplitude of the 300 ms-reference stimulus $L_{\text{\_\{ref\}}}$ (in CU). This procedure allowed a more accurate balancing and has been used by McKay et al. (2003) and Adel et al. (2017).

(1) $$L_{\text{\_\{probe\}}}=L_{\text{\_\{ref\}}}+\frac{d_{\text{\_\{noninverted\}}}+d_{\text{\_\{inverted\}}}}{2}$$

In both cases (noninverted and inverted), the probe stimulus had a starting value that randomly varied above (between +5 % and +10 % DR) or below (between −10 % and −5 % DR) the estimated level. This procedure was chosen to minimize the bias when adjusting to a standard stimulus (Poulton, 1989: chap. 3). In the first blocks, the best estimation of the probe was to take the same level of the DR that was used for the reference stimulus. In the specific cases where no MALs could be obtained, care was taken to first start from below so that the second run could start from a louder level. As the BALs were measured sequentially, randomization of electrode × rate × duration conditions was only done within each block of Figure 3 (blocks ⓪ to ④).

### Analyses

For each subject and condition, the mean over the corresponding THR/MAL measurements was taken. The DRs were calculated by dividing the mean THR by the mean MAL and then converted into dB. Equation 1 was used to obtain a single BAL value from the four loudness balancing measurements. For averaging over different subjects, means were calculated. Regular standard deviations were calculated to show variability within and between subjects to allow easier comparisons to other publications.

Conventional nonlinear least squares estimation was used to fit THR, BAL, and MAL amplitudes to a power-law as a function of the number of pulses. All calculations were done with amplitudes in CU and only transformed into dB re 1 CU for visual display and comparisons.

#### Statistical Analysis

A repeated measures analysis of variance (RM ANOVA) was applied when calculating the effects of several factors on the results. An alpha-level of 0.05 was used. Bonferroni correction was used to correct for multiple testing.

The slopes of the fitted power-laws were tested with a three-way (three within-subject factors) RM ANOVA with the factors Measurement (THR, BAL), Rate (1,500; 18,000 pps), and Electrode (apical, basal). MAL slopes were left out of the RM ANOVA since MALs could often not be measured for very short durations without exceeding the compliance limit, which, in turn, might result in an underestimation of MAL slopes. Furthermore, MALs might not have an equal loudness across durations. Eleven out of 12 subjects were included in this analysis due to some BAL measurements out of compliance for S2.

The statistical power of the RM ANOVA was tested by conducting a power analysis in R, also available as an online tool (Superpower’s Power Shiny App, https://shiny.ieis.tue.nl/anova˙power/) developed by Lakens & Caldwell (2021). The Superpower package estimates the power of factorial ANOVA designs based on Monte Carlo simulations of the experimental design. We ran a Monte Carlo simulation ( $n_{\text{\_\{simulation\}}}=2000$ , $n_{\text{\_\{subjects\}}}=11$ ) with all three factors Measurement (THR, BAL), Rate (1,500; 18,000 pps), and Electrode (apical, basal), correcting for multiple testing (Bonferroni). The main effects of Measurement and Rate showed sufficient power (100%) and very large effect sizes ( $\eta^{2}\gg 0.14$ ).

## Results

### Effects of Duration, Rate and Electrode

Figure 1 shows exemplary results for two subjects. THR, BAL, and MAL amplitudes decreased with increasing duration (or equivalently, increasing number of pulses). It is also visible that sometimes, for very short durations, no data points for the MAL could be obtained because of the compliance limit. In panel A (S1l), the fitted curves (solid lines) of THR and MAL appear to be almost parallel, while the BAL curve is shallower than the other two. In contrast, in panel B (S7), MAL and BAL seem to drop similarly for increasing duration, whereas the THR curves have different slopes. In general, BAL data needs to be interpreted with caution as many subjects reported having problems comparing the loudness of short and long stimuli precisely.

Figure 2 shows the mean over all subjects’ results for the THR, BAL, and MAL measurements as a function of duration (or equivalently, number of pulses) for each of the 4 electrode × rate combinations. Just as in the exemplary data (Figure 1), decreased THR, BAL, and MAL amplitudes were observed with increasing stimulus duration (TLI), especially at the higher rate. A reduction in amplitude could also be observed for the higher rate when comparing across stimuli of equal duration (MPI). On average, no systematic effects of the electrode location on the adjusted amplitudes was observed.

### Temporal Loudness Integration

Several phenomenological models for (acoustic) TLI have been proposed in the past (Garner & Miller, 1947; Green et al., 1957; Hughes, 1946; Plomp & Bouman, 1959). Of those, the power function (which appears as a linear dependency on double-logarithmic axes) is one of the simplest options to fit normal-hearing TLI data. The decision to fit this function to our data was not only based on its simplicity but because it also allowed for a direct comparison between the slopes of the fitted functions. On the downside, we found that our data did not cover long enough durations to determine the critical duration, after which loudness does not vary with duration anymore (Munson, 1947; Stevens & Hall, 1966). However, from visual inspection, it seemed like this saturation point was only reached in a few subjects for specific conditions. This possible critical duration was ignored in the following analyses, as it was alno not consistently found in the acoustic literature at similar durations (Florentine et al., 1988; Gerken et al., 1990).

THR, BAL, and MAL current amplitudes ( I in CU) were fitted as a function of the number of pulses for each subject and electrode × rate condition to a power-law of the form

(2) $$I(N)=I_{1}\cdot N^{m}.$$

The parameter $I_{1}$ (in CU) is the current amplitude for single pulse stimulation and m represents the slope of the decreasing amplitudes for increasing number of pulses N . Fits of the THR data worked quite well, with an average of $R^{2}=0.98\pm 0.02$ . Data obtained in the other measurements were fitted with $R^{2}=0.91\pm 0.04$ (BAL) and $R^{2}=0.91\pm 0.16$ (MAL). Further details about the goodness of fit obtained from fitting THR, BAL, and MAL amplitudes to a function of the number of pulses (equation 2) are specified in Table 3, where the means and standard deviations of the fitting parameters $I_{1}$ and m are also listed. Exemplary fittings of the power function are included in Figure 1. In this figure, the data points of subject S1l (panel A) and S7 (panel B) are shown together with three solid lines corresponding to the fits of the different measurements (THR, BAL, MAL). Additionally, the dotted lines show the fits for the THR data if the slope m would correspond to −20∕3 dB per tenfold increase in duration ( m=−0.33 ) found in normal hearing.

**Table 3.** Results of Data Fits with equation 2 for the Parameters $I_{1}$ and m (slopes).

Measurement	Electrode	Rate (pps)	$R^{2}$	$I_{1}$ (CU)	m
THR	apical	1,500	0.98±0.02	452.66±153.03	−0.16±0.07
THR	apical	18,000	0.99±0.01	458.73±151.37	−0.27±0.05
THR	basal	1,500	0.97±0.02	451.10±113.19	−0.19±0.04
THR	basal	18,000	0.98±0.01	455.58±111.20	−0.28±0.05
MAL	apical	1,500	0.83±0.16	1041.43±273.90	−0.09±0.04
MAL	apical	18,000	0.94±0.04	1277.85±466.21	−0.14±0.03
MAL	basal	1,500	0.92±0.07	1082.88±230.24	−0.11±0.02
MAL	basal	18,000	0.95±0.03	1230.97±405.33	−0.13±0.05
BAL	apical	1,500	0.85±0.16	788.47±271.53	−0.09±0.02
BAL	apical	18,000	0.95±0.02	771.12±253.19	−0.16±0.03
BAL	basal	1,500	0.91±0.06	756.44±146.60	−0.11±0.03
BAL	basal	18,000	0.94±0.04	861.28±229.55	−0.17±0.04

*Note*. Means ± standard deviations (SD) across subjects. MAL = maximum acceptable level; THR = threshold

**Figure 4.**
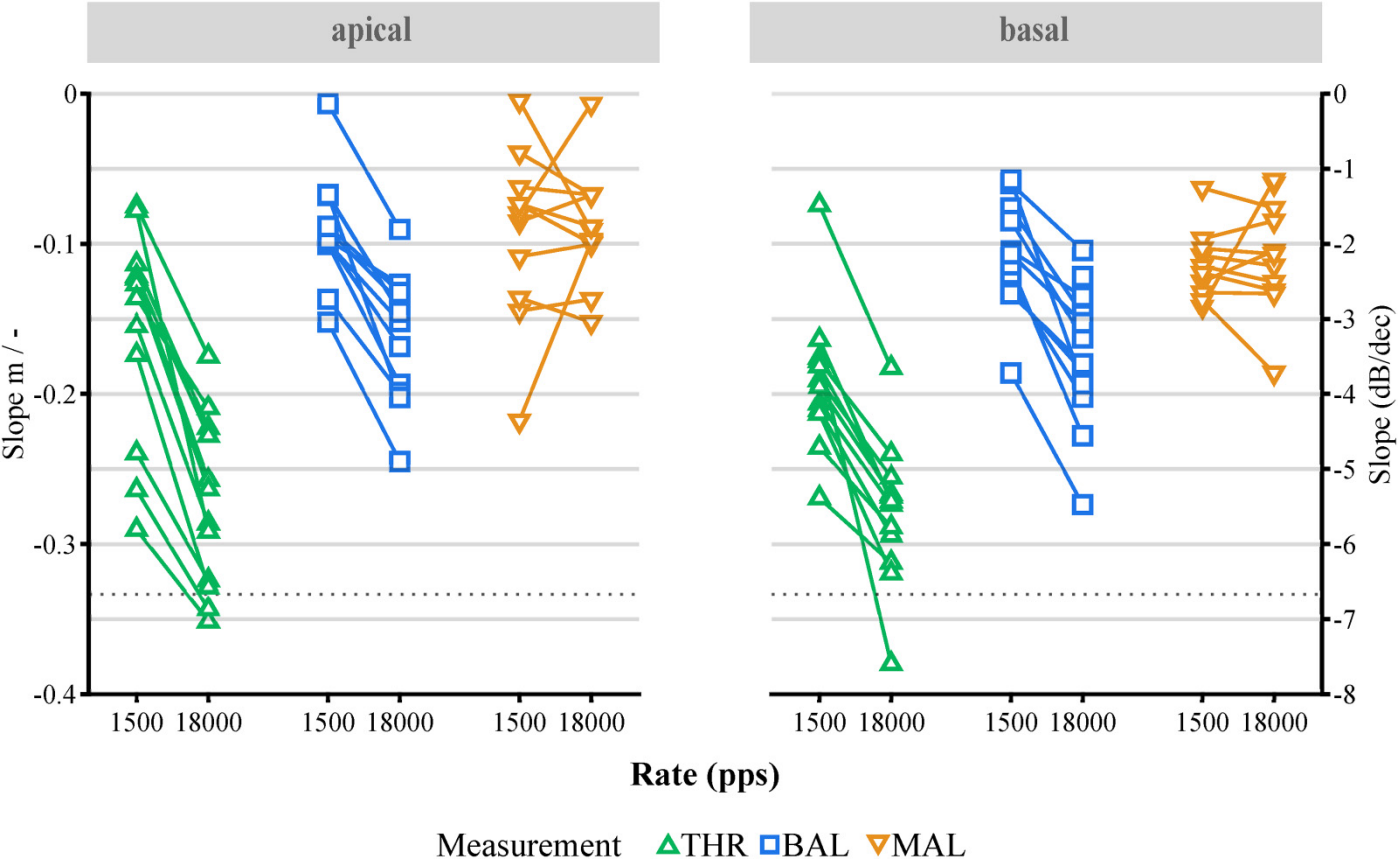
Slopes m of the fitted (equation 2) THR, loudness balancing (BAL), and MAL TLI curves for each rate and electrode location. The right axis shows the fitted parameter m (equation 2) and the left axis the slope expressed in dB per decade. Individual symbols represent the slopes of the fits for a single subject, connected by a line to visualize the effect of the stimulation rate. The dotted line represents the slope of −20∕3 dB per decade ( m=−0.33 ) found in normal hearing. Left: Apical electrode. Right: Basal electrode. MAL = maximum acceptable level; THR = threshold; TLI: temporal loudness integration.

The slopes of the fitted THR, BAL, and MAL functions for every subject for each rate, and electrode are shown in Figure 4. Within one measurement (THR, BAL, MAL) and electrode position (apical, basal), the data points corresponding to the same subject are connected by a line to better visualize the effect of stimulation rate on the slopes of the TLI curves. An RM ANOVA on the slope of the fitted functions testing the effects of Measurement (THR, BAL), Rate (1,500; 18,000 pps), and Electrode (apical, basal) showed a highly significant effect of Measurement [ $F(1,10)=42.29,p<0.001,\eta^{2}=0.81$ ]. On average, the slopes at THR level were steeper than those at BAL ( p<0.001 , $M_{Diff}=0.09,95\%-CI[0.06,0.12]$ ) and MAL (Figure 4). The dissimilar slopes between THR and MAL resulted in larger DRs with increasing duration. In addition, TLI slopes were significantly affected by the stimulation Rate [ $F(1,10)=127.46,p<0.001,\eta^{2}=0.93$ ]. Figure 4 shows how THR and BAL TLI curves become steeper for every subject at the higher rate ( p<0.001 , $M_{Diff}=0.09,95\%-CI[0.07,0.11]$ ). Increasing the stimulation rate had a larger effect on THR and BAL slopes than on MAL slopes (Figure 4). At the higher rate, MALs became steeper ( $\Delta {\bar{m}}_{MAL}=0.05\pm 0.02$ ) to a smaller extent compared to THR ( $\Delta {\bar{m}}_{THR}=0.10\pm 0.03$ ) and BAL ( $\Delta {\bar{m}}_{BAL}=0.07\pm 0.02$ ). This dissimilar increase in THR and MAL slopes upon increasing the stimulation rate resulted in larger DRs for the higher rate (Figure 2). The Electrode did not have a significant effect on the slopes of the fitted curves [ F(1,10)=2.37,p=0.16 ]. All remaining and higher order interactions did not reach significance level.

The slope of the fitted functions and the absolute THR amplitudes (in CU) correlated relatively high (Spearman’s ρ≥0.68 , p≤0.01 ) for the 18,000 pps condition for durations equal to and higher than 3 ms at both electrode locations. The lower rate (1,500 pps) only exhibited a significant strong correlation (Spearman’s ρ=0.70 , p=0.008 ) for the 300 ms duration at the basal electrode.

## Discussion

In investigations with CI users, often a large variability between the individual subjects’ performance is observed. This was also the case in the present experiment, where a large variability of absolute THR, BAL, and MAL current amplitudes is apparent. This can partly be explained by different progression of diseases (including degeneration of the distal parts of SGNs), different levels of training with the CI due to differing time spans since implantation, residual hearing of the nonimplanted side, or a dissimilar positioning of the electrode array in the cochlea, among different CI users. For this reason, we calculated the slopes of TLI on an individual level. Nonetheless, we measured effects of stimulation duration and stimulation rate that were common to all participants, even if the absolute current amplitudes differed.

### Effects of Duration, Rate and Electrode

Overall, BAL and MAL curves in Figure 2 shared similar slopes, whereas the THR curve revealed steeper slopes when compared to the other two. This is reflected in the fitted slope parameters (Table 3, Figure 4). The main effect of stimulation rate (lower amplitudes for the higher rate, MPI) on threshold is in line with previously published results – if not somewhat higher. For pulse trains of 300 ms duration, we found on average 12.0 dB lower threshold amplitudes when stimulating with 18,000 pps instead of 1,500 pps (rate increased by a factor of 12), which is equivalent to a decrease of 3.3 dB per doubling of the rate.

Comparable but slightly lower values were found by Carlyon et al. (2015), with a decrease of 7.7 dB for 400 ms pulse trains after increasing the rate from 500 to 3,500 pps, equivalent to a threshold decrease of 2.7 dB per doubling of the rate. Kreft et al. (2004) found a reduction in threshold of 2.4 dB per doubling of the rate between 200 and 6,500 pps, with a reduced effect beyond 3,250 pps for 200 ms pulse trains. In Zhou et al. (2015), detection thresholds for 250 ms pulse trains showed a median decrease of around 2.6 dB per doubling of the rate for rates higher than 1,000 pps. Lower amplitudes caused by higher rates (MPI) might be attributed to the effects of facilitation. With small interpulse intervals (equivalent to the rates above 1,000 pps), when a single fiber is in its absolute/relative refractory period, a subsequent pulse may still be able to elicit action potentials in other fibers. Even when the subthreshold pulses cannot cause an action potential, they may facilitate it for the subsequent pulses to cause the fiber to fire (Boulet et al., 2016). This facilitative effect has been suggested to be due to the residual partial depolarization of the cell membrane caused by subthreshold pulses (Middlebrooks, 2004).

As also the literature suggested (e.g. Zhou et al., 2012), in this study no systematic effect of stimulation electrode along the tonotopic axis was found for THR amplitudes. Nevertheless, some subjects showed markedly lower THRs for one of the electrodes. An extreme case was S7, for whom THRs at the basal electrode were at least 8.9 dB higher than at the apical electrode, for both of the tested rates (Figure 1B).

MAL decreased, on average, by 4.8 dB between the two rates, or 1.3 dB per doubling of rate (300 ms pulse trains), comparable to the results in Kreft et al. (2004) of 1.2 dB per doubling, and somewhat lower than those in Skinner et al. (2000) of around 1.7 dB per doubling between 1,200 and 2,400 pps.

### Temporal Loudness Integration

In electric hearing, differences between threshold and suprathreshold curves have been described by McKay and McDermott (1998). They described that at higher levels, an increase in stimulus amplitude leads to a larger increase in aggregated stimulation than at lower levels. This was investigated by measuring loudness changes evoked by varying the interpulse interval between two pulses in a pair—which was itself repeated at 50 Hz. If it is possible to extend their findings to longer pulse trains, and it is assumed that similar levels of excitation in the nerve lead to similar loudness percepts, then it would be expected that smaller amplitude reductions are necessary at higher levels to compensate for longer pulse trains. This would imply steeper TLI curves at threshold than at higher levels, as was seen in this experiment (Figure 4).

A study with normal-hearing subjects revealed nonmonotonic level dependencies on the slope of TLI curves (Florentine et al., 1996). Both 1 kHz tones and white noise (WN) were investigated at 5 ms, 30 ms, and 200 ms. The strongest TLI was found at medium levels for both tones (around 56 dB SPL) and WN (around 76 dB SPL). Interestingly, they attribute this effect to properties of the loudness function related to the basilar membrane mechanics, which should not play any role in our subjects. In the present study, slopes at threshold were steeper ( p<0.001 ) ( ${\bar{m}}_{\text{\_ \{THR\}}}=-0.23\pm 0.07$ ) than at moderate ( ${\bar{m}}_{\text{\_\{BAL\}}}=-0.13\pm 0.04$ ) or maximum loudness ( ${\bar{m}}_{\text{\_\{MAL\}}}=-0.12\pm 0.04$ ). As in the results of Gerken et al. (1990), who also used a power-law function for fitting, we do not have to vary the exponent with duration (and in contrast to the results of e.g. Green et al., 1957). At least for THR amplitudes up to 300 ms, goodness of fit is excellent with an overall mean of $R^{2}=0.98\pm 0.02$ .

Just like reported in other studies, our data contained large interindividual variability. Regarding the slopes of the fitted THR functions, results at conditions of e.g. basal positions at 1,500 pps varied from $m_{\text{\_\{THR\}}}=-0.07$ for subject S7 to $m_{\text{\_\{THR\}}}=-0.27$ for subject S4r. The mean of ${\bar{m}}_{\text{\_\{THR\}}}=-0.17\pm 0.06$ for the low rate and ${\bar{m}}_{\text{\_\{THR\}}}=-0.28\pm 0.05$ for the high stimulation rate can be translated to changes of −3.49±1.19 and −5.52±1.00 dB per decade, respectively. The slope for the high stimulation rate is closer to the amplitude decrease of 6.67 dB per decade for acoustic hearing reported by Heil et al. (2017), whereas the curve for the lower rate stimulation is not as steep.

Already Donaldson et al. (1997) reported much shallower slopes for CI users when compared to normal-hearing listeners, whose slopes are at about −2  or −3 dB per doubling of duration (see also Shannon, 1983). At a relatively high stimulation rate of 10,000 pps, Saeedi and Hemmert (2020) reported a TLI slope of −1.30 dB per doubling the stimulation duration for CI users, which is equivalent to a slope of −4.32 dB per decade. This slope falls properly between the two slopes of −3.49 and −5.52 dB per decade found in this study for the rates of 1,500 and 18,000 pps, respectively. It seems that only very high stimulation rates, the slopes of CI users become more similar, i.e. as steep, to those of normal-hearing listeners. However, this similarity is more surprising than expected, given that acoustic hearing involves large DR compression by the inner ear mechanics, which is completely missing in electric hearing. The DR in electric hearing is by orders of magnitude lower than for acoustic stimulation and therefore, at the higher rate, the amount of TLI even exceeds the DR. At 18,000 pps, single-pulse (SP) THRs were, on average, 1.37±2.55 dB above the corresponding 300-ms MCLs. This is clearly visible for subject S7 (Figure 1) at 18,000 pps at the basal electrode and is reflected in the mean over all subjects (Figure 2) at both electrode locations (apical: ${\bar{THR}}_{SP}=52.44\pm 2.84\;dB\;re\;1\;CU>{\bar{MCL}}_{300ms}=51.01\pm 3.63\;dB\;re\;1\;CU$ ; basal: ${\bar{THR}}_{\text{\_\{SP\}}}=52.64\pm 1.91dB\;re\;1\;CU>{\bar{MCL}}_{300ms}=51.33\pm 3.02dB\;re\;1\;CU$ ). Even though at 1,500 pps, single-pulse THRs did not exceed the DR of the corresponding 300 ms stimuli, they only were, on average, 2.77±1.73 dB below the 300-ms MCLs. This means that very short signals are hardly audible for typical fitting procedures (1,500 pps).

### Dynamic Range

The DR increased with increasing stimulation rate for all durations, but even more for longer than for shorter stimulus durations. For the 300 ms pulse trains, an increase in DR of 7.36±3.16 dB was observed. The general increase can be explained by the fact that especially threshold amplitudes are lowered by increasing the rate, but not so much the amplitudes of measurements at the higher stimulation levels (Figure 4). This results in a larger DR at higher rates compared to lower rates (Kreft et al., 2004; Pfingst et al., 2007). The same has been reported by McKay and McDermott (1998).

Bonnet et al. (2012) reported an increase in DR of 1.3 dB for a doubling of the stimulation rate. Our values suggest 2.05 dB for a doubling of the rate. Since the rates we compared (1,500 and 18,000 pps) are much higher than theirs (774 and 3,868 pps), there could be an additional influence of the effect of rate on THR and MAL for rates higher than 3,868 pps. The increase of DR in our data is also much larger than the one found by Zhou et al. (2012). They report an increase in DR of 1.19 dB for a doubling of the stimulation rate, for increasing the rate up to 5,000 pps.

The fact that for both rates the DR increased even more for long durations is reflected by the differences in slopes for the two measures, where THRs decreased with a steeper slope of −0.23, compared to the slope of −0.12 found for MALs (Figure 4). This is in line with the literature, which reported shallower slopes for maximum amplitudes (also called *C-levels*) when compared to those for the threshold amplitudes (also called *T-levels*) (Zhou et al., 2012).

For CI fitting in the clinics, only single electrode DRs are set. Since neurons in the cochlea also experience stimulation of neighboring electrodes due to wide electrical current spread, their effective stimulation occurs in bursts, where the burst rate is the single electrode rate times the number of stimulated electrodes (as the high rate in our measurements). For this high rate, the THR values are lower and the DR higher than currently set in clinical CI fittings. Accurate consideration of the TLI at different rates during the fitting process could therefore result in a better usage of the limited DR available in electric hearing and, therefore, could be used to better CI performance.

## Conclusions

In the present work, we measured TLI curves in CI users at three different levels, at a moderate and a very high rate, and at two positions in the electrode array. In general, an increase in stimulation rate lowered the current amplitudes at all loudness levels (MPI), and this effect of rate was stronger at THR than at MAL stimulation. This led to an increased DR for the higher rate that seems consistent, if not higher, than those found in the literature.

We also showed that a power-law function can describe the TLI curves in CI users with high accuracy, as also suggested in normal-hearing listeners. The slopes of the TLI curves were also steeper for THR than for BAL and MAL stimuli. The effect of rate itself was stronger on the slopes of THR when compared to those of MAL levels. In comparison to the lower rate, the higher rate led to a steeper THR slope, where, surprisingly, the slope of the TLI curves approached that reported in normal-hearing listeners.
